# Effect of cinacalcet availability and formulary listing on parathyroidectomy rate trends

**DOI:** 10.1186/1471-2369-14-100

**Published:** 2013-05-03

**Authors:** Jean-Philippe Lafrance, Héloïse Cardinal, Martine Leblanc, François Madore, Vincent Pichette, Louise Roy, Jacques Le Lorier

**Affiliations:** 1 Centre de recherche Hôpital Maisonneuve-Rosemont, Montréal, Canada; 2 Département de Médecine, Université de Montréal, Montréal, Canada; 3 Service de néphrologie, Hôpital Maisonneuve-Rosemont, 5415, boul. de l’Assomption, Montréal, QC, H1T 2M4, Canada; 4 Service de néphrologie, Centre hospitalier de l’Université de Montréal, Montréal, Canada; 5 Service de néphrologie, Hôpital du Sacré-Cœur de Montréal, Montréal, Canada; 6 Centre de recherche Centre hospitalier de l’Université de Montréal, Montréal, Canada

**Keywords:** Parathyroidectomy, Hyperparathyroidism, Secondary, Calcimimetic agents, Renal dialysis, Epidemiologic studies

## Abstract

**Background:**

Recent trends in parathyroidectomy rates are not known. Our objective was to investigate the trend in parathyroidectomy rates between 2001 and 2010, and to evaluate if the availability and reimbursement of cinacalcet modified that trend.

**Methods:**

Using a provincial administrative database, we included all adult patients receiving chronic dialysis treatments between 2001 and 2010 (incident and prevalent) in a time series analysis. The effect of cinacalcet availability on parathyroidectomy bimonthly rates was modeled using an ARIMA intervention model using different cut-off dates: September 2004 (Health Canada cinacalcet approval), January 2005, June 2005, January 2006, June 2006 (date of cinacalcet provincial reimbursement), and January 2007.

**Results:**

A total of 12 795 chronic dialysis patients (mean age 64 years, 39% female, 82% hemodialysis) were followed for a mean follow-up of 3.3 years. During follow-up, 267 parathyroidectomies were identified, translating to an average rate of 7.0 per 1000 person-years. The average parathyroidectomy rate before cinacalcet availability was 11.4 /1000 person-years, and 3.6 /1000 person-years after cinacalcet public formulary listing. Only January 2006 as an intervention date in the ARIMA model was associated with a change in parathyroidectomy rates (estimate: -5.58, p = 0.03). Other intervention dates were not associated with lower parathyroidectomy rates.

**Conclusions:**

A reduction in rates of parathyroidectomy was found after January 2006, corresponding to cinacalcet availability. However, decreased rates may be due to other factors occurring simultaneously with cinacalcet introduction and further studies are needed to confirm these findings.

## Background

Secondary hyperparathyroidism is a common and serious disease that develops early in chronic kidney disease and continues to progress after subjects reach dialysis. A primary consequence of secondary hyperparathyroidism is the development of renal osteodystrophy, which may manifest as fractures, bone pain and avascular necrosis [1]. Prevention and treatment of secondary hyperparathyroidism remain a continuous challenge for the nephrologist, despite availability of phosphate binders, active vitamin D analogues and more recently, cinacalcet.

Cinacalcet, a calcimimetic, was approved in March 2004 in the United-States (US) and in September 2004 in Canada. Parathyroidectomy (PTX) is primarily reserved for symptomatic patients with elevated parathyroid hormone (PTH) levels despite optimal medical therapy.

While cinacalcet has been shown in trials to reduce PTH levels [2] and to lower incidence of PTX [3,4], it remains unclear how availability of this new drug influenced PTX rate trends in a real-world setting. While PTX may be avoided initially by using cinacalcet, the surgery may only be delayed to a later time, which would not be observed in a short-term study. Moreover, efficacy of cinacalcet may be limited in some patients by its gastrointestinal adverse effects, which may be more frequent in real-world patients compared to selected patients enrolled in a randomized-controlled trial. In a recent placebo-controlled trial, 18% of patients in the cinacalcet group discontinued the drug because of adverse events and 7% underwent a PTX [4].

Rates of PTX varied considerably between 1992 and 2007 [5-8]. These fluctuations were attributed to availability of new therapeutic options (vitamin D analogs and non-calcium-containing phosphate binders) and publication of practice guidelines [5]. Only one study evaluated PTX rate trends after availability of cinacalcet, which showed a decreasing PTX rate until 2005 followed by an increase in rates in 2006–2007. However, this study was limited to 2007 (less than three years after cinacalcet marketing) [5]. In addition, the effect of formulary listing of cinacalcet for reimbursement on PTX rates has not been studied.

The aim of this study was to investigate the trend in annual PTX rates between 2001 and 2010, and evaluate if availability and formulary listing of cinacalcet modified that trend.

## Methods

### Study population and data sources

This study was conducted using the *Régie de l’assurance maladie du Québec* (RAMQ) database in the province of Québec, Canada. This universal provincial health services administrative database includes all visits, diagnosis codes (*International Classification of Diseases, Ninth* then *Tenth Edition* (ICD-9, ICD-10)) and procedures (*Canadian Classification of Diagnostic, Therapeutic and Surgical Procedures* (CCP) and *Canadian Classification of Health Interventions* (CCI)) during in- or outpatient medical encounters for virtually all eight million residents of Québec, Canada. Admission and discharge dates, primary and secondary diagnoses (coded using ICD-9 or ICD-10 after 2006), and procedures during a hospitalization were obtained from the hospital discharge summary database. In addition, information on drugs dispensed to all individuals age 65 years and older, individuals on welfare and workers insured by the provincial drug plan is captured within the database. Validation of RAMQ data has shown high accuracy for a number of conditions [9-12].

### Study sample

All adults (≥18 years old) who were on chronic dialysis (hemodialysis or peritoneal dialysis) between January 1, 2001 and December 31, 2010 were included. Chronic dialysis was defined as at least 90 days of dialysis, and therefore patients with less than 90 days of follow-up were excluded. Both incident (new patients initiating dialysis during the study period) and prevalent (patients already on dialysis at the beginning of the study) patients were included. Patients with a prior history of PTX (same codes as for outcome) or kidney transplant (ICD-9: V42.0; ICD-10: Z94.0; RAMQ: 06221, 06222, 06223) were excluded, using a two-year look back period (*e.g.* January 1999 for patients on dialysis on January 2001). Patients were censored at PTX, death, kidney transplantation, emigration out of the province, or end of the study (December 31, 2010).

### Outcome

PTX was the primary outcome and was identified through hospital discharge summary procedure codes (CCP: 19.71, 19.72; CCI: 1FV83, 1FV87, 1FV89) and RAMQ physician claim code (6186, 6185, 6181).

### Intervention period

In Canada, a new drug cannot be marketed before being approved by Health Canada, which evaluates efficacy and safety of the drug. After this approval, all patients may receive the drug, but it will not be reimbursed, except if the patient is covered by a private insurance company reimbursing that particular drug. Policies for reimbursement of drugs vary by provinces. For the provincial drug plan in Québec, decision to reimburse or not a drug is recommended by a provincial committee from the *Institut national d'excellence en santé et en services sociaux (INESSS),* which evaluates its cost-effectiveness ratio and possible financial burden. Cinacalcet was approved by Health Canada on September 2004 and became available for sale in the province of Québec shortly thereafter. However, cinacalcet was not reimbursed via the provincial formulary until June 2006, and was only reimbursed by private insurance companies for a few patients who had private insurances coverage (less than 22% annually). However, cinacalcet was provided by Amgen Canada from September 2004 until June 2006 for few selected patients with severe secondary hyperparathyroidism. Of note, once listed on the provincial formulary, cinacalcet was available only for patients meeting specific criteria: 1) at least two PTH values above 88 pmol/L (829 pg/mL) in a three-month period despite optimal medical therapy with vitamin D and phosphate binders; and 2) at least one of the following: serum calcium ≥2.54 mmol/L (10.2 mg/dL), serum phosphorus ≥1.78 mmol/L (5.5 mg/dL), calcium-phosphorus product ≥4.5 mmol^2^/L^2^ (55 mg^2^/dl^2^), or bone symptoms. For cinacalcet to be reimbursed for a given patient, the prescriber would need to obtain a prior authorization from the provincial drug plan by filling a form detailing those criteria.

### Statistical analysis

Autoregressive integrated moving average (ARIMA) intervention models were used to analyze the effect of cinacalcet availability on PTX rates. The ARIMA intervention model is a specific time series model. It controls for trend and seasonal variation and allows for the insertion of input variables to check and evaluate the effects of interventions: cinacalcet availability in this case. Because it produced the most efficient number of points for this analysis, bimonthly PTX incidence rates were calculated throughout the study duration. Since the intervention period (availability of cinacalcet) was not a single point in time (it became available progressively from September 2004 to June 2006 as described above), different *a priori* defined dates in that period were used in the model: Health Canada approval date (September 2004), Quebec reimbursement (June 2006), in-between dates (January 2005, June 2005, January 2006), and six-month post-reimbursement (January 2007). The adequate candidate model was chosen according to published guidelines (parameters significance, graphical evaluation of residuals, and Akaike Information Criterion) [13,14].

As a sensitivity analysis, rates of PTX pre and post introduction of cinacalcet were compared using a Poisson regression. In opposition to ARIMA models, Poisson regression does not account for the dependence between measures seen in our situation (a patient may be present in different intervals), but its results are more intuitive and may help validate results from ARIMA. For this analysis, two periods were defined: 1) a pre cinacalcet period from January 1, 2001 until August 31, 2004, and 2) a post cinacalcet period from June 30, 2006 until December 31, 2010. Time between the two periods was not considered in this analysis. As a second sensitivity analysis, ARIMA and Poisson regression analyses were redone by excluding periods of time where patients were not covered by the RAMQ drug plan.

Because we expected that important patient characteristics such as age, sex, and incident/prevalent status would change with time [15,16], and that these factors are related to PTX for secondary hyperthyroidism [17], we plot PTX rates stratified by those characteristics.

Since cinacalcet is not the only factor that changed over the study period, phosphate binders (sevelamer, lanthanum, calcium-containing, and magnesium and/or aluminium-containing) and vitamin D use was also measured. Annual medication use was calculated by dividing the number of patients with at least two drug dispensations in a given year by the number of patients at least partially covered by RAMQ drug plan in that given year. In order to ensure prescriptions were being fulfilled, two dispensations were requested for each patient. The average daily dose for selected medication was calculated including: calcium-containing binders, sevelamer, lanthanum, alfacalcidol, calcitriol, and cinacalcet. Total dose of phosphate binders was calculated using relative phosphate-binding coefficients in order to transform doses of sevelamer and lanthanum to calcium mg equivalent: 1.0 for calcium carbonate, 0.7 for sevelamer and 2.0 for lanthanum [18]. Alfacalcidol and calcitriol are by far the two frequently used activated vitamin D products in Québec.

For all analyses, a p-value of less than 0.05 was considered statistically significant. All analyses were done using SAS 9.2 (Cary, North Carolina).

### Ethical considerations

This study was approved by the Government of Québec ethics committee (*Commission d’accès à l’information*) and Maisonneuve-Rosemont Hospital ethics committee, and informed consent was waived.

## Results

**Figure 1:**
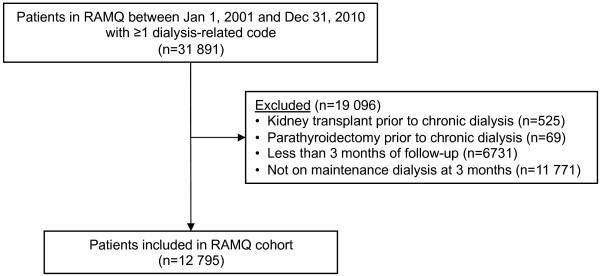
Derivation of cohort.

There were 12 795 patients on maintenance dialysis in Québec between January 1, 2001 and December 31, 2010. Derivation of the study cohort is detailed in Figure 1. At the beginning of follow-up, the mean age was 63.9 years (SD = 14.6), females represented 39%, and 82% of patients were on hemodialysis. The mean follow-up was 3.3 years (SD = 2.6). Most patients had at least part of follow-up covered by the drug plan (partly for 19.5%, and full for 64.0%), as only 16.5% had only private coverage during follow-up. Almost half of all patients (49%) died during follow-up and 15% were transplanted. As shown in Table 1, patient age increased with time, while proportions of incident patients and of patients on peritoneal dialysis decreased with time.

**Table 1:** Patients’ characteristics

	**2001**	**2002**	**2003**	**2004**	**2005**	**2006**	**2007**	**2008**	**2009**	**2010**
	**(%)**	**(%)**	**(%)**	**(%)**	**(%)**	**(%)**	**(%)**	**(%)**	**(%)**	**(%)**
*Age (years)*										
<55	28.3	27.3	26.9	25.3	24.4	23.8	23.1	22.7	22.6	22.2
55-64	20.7	21.4	21.2	22.1	22.2	22.2	22.2	22.2	22.1	22.0
65-75	30.1	29.8	29.6	29.9	30.1	29.9	29.9	29.5	29.5	29.8
≥75	20.9	21.5	22.3	22.7	23.3	24.2	24.8	25.6	25.8	26.1
*Sex*										
Female	39.7	40.5	39.9	40.3	39.9	40.4	41.0	40.8	41.3	40.5
Male	60.3	59.5	60.1	59.8	60.1	59.6	59.0	59.2	58.7	59.5
*Dialysis modality*										
Hemodialysis	77.0	78.3	80.2	81.7	82.1	83.4	84.1	84.1	84.5	85.0
Peritoneal dialysis	23.1	21.7	19.8	18.3	17.9	16.6	15.9	15.9	15.5	15.1
*Incidence vs. prevalence*										
Prevalent	55.3	55.7	57.3	58.3	59.6	60.2	61.4	61.7	62.4	66.7
Incident	44.7	44.3	42.7	41.7	40.4	39.9	38.6	38.3	37.6	33.3

**Figure 2:**
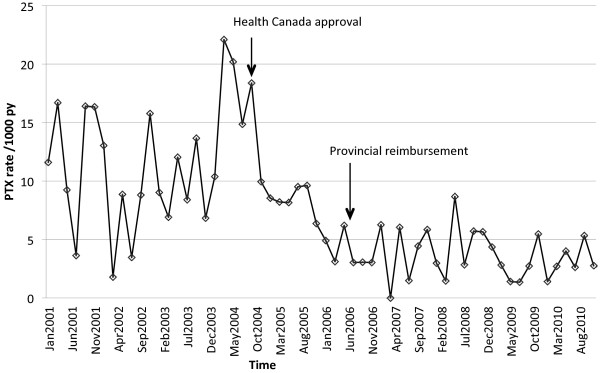
**Bimonthly parathyroidectomy rates.** Abbreviations: PTX, parathyroidectomy; py, person-year.

**Figure 3:**
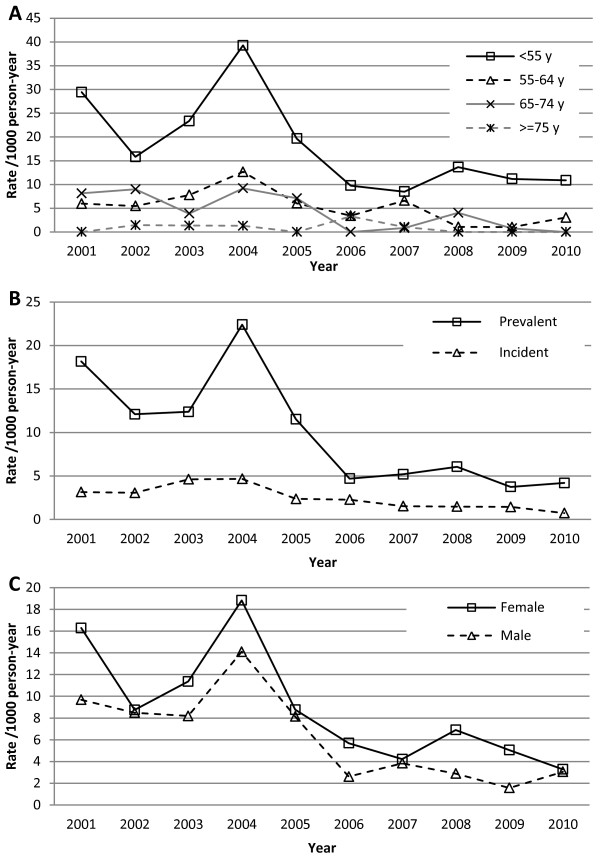
**Parathyroidectomy rates by age, sex, and incidence/prevalence.** Panel **A**. Parathyroidectomy rates by age categories. Age is calculated at cohort entry. Panel **B**. Parathyroidectomy rates by sex. Panel **C**. Parathyroidectomy rates for incident and prevalent patients. See text for definition of incident patients. Abbreviations: PTX, parathyroidectomy; py, person-year.

During follow up, 267 PTX were identified, which translated to an average rate of 7.0 per 1000 py. Bimonthly rates of PTX are given in Figure 2. The average rate before cinacalcet approval was 11.4 /1000 py and was 3.6 /1000 py after cinacalcet reimbursement. The best fitting ARIMA model did not include a trend term over time. In other words, rates did not appear to increase or decrease over time except at the intervention point. Only January 1, 2006 as an intervention date was statistically significant (estimate = −5.58; p = 0.03). Other intervention dates were not associated with a reduction in PTX rates: September 2004, estimate = −2.98, p = 0.3; January 2005, estimate = −5.04, p = 0.07; June 2005, estimate = −4.51, p = 0.09; June 2006, estimate = −3.72, p = 0.2; January 2007, estimate = −2.53, p = 0.4. Using a Poisson regression as a sensitivity analysis, rates were 68% lower after cinacalcet introduction compared to before (incidence rate ratio = 0.32; 95% confidence interval: 0.23-0.42; p < 0.001). Results for the ARIMA and Poisson analyses were similar when time periods not covered by the drug plan were excluded, except that June 2005 became statistically significant in addition to January 2006. As expected, PTX rates appeared higher for young patients than older patients, and in prevalent patients compared to incident patients (Figure 3). PTX rates did not differ by gender. However, trends of PTX rates were similar across strata.

**Figure 4:**
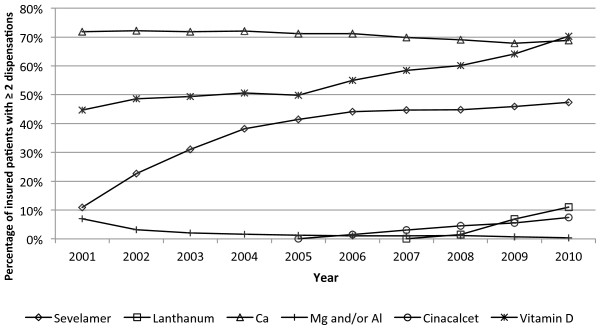
**Trends in selected medication use.** Note: Vitamin D category includes vitamin D and analogues. Abbreviations: Ca, calcium-containing phosphate binders; Mg, magnesium-containing phosphate binders; Al, aluminum-containing phosphate binders.

Trends in medication use are given in Figure 4 and average daily doses are shown in Table 2. The proportion of patients using calcium-containing phosphate binders remained stable over time, but the average daily dose decreased with time. On the contrary, the proportion of patients using sevelamer increased until 2005 then remained fairly stable. The average daily dose of sevelamer followed the same pattern of use. Overall, the average daily combined dose of phosphate binders increased with time. The percentage of patients on vitamin D was stable until 2005, and then started to rise. The average daily dose of alfacalcidol increased only very slowly however, and calcitriol daily dose remained stable. Use of cinacalcet increased steadily after 2006, while lanthanum was introduced in 2007. For both, the average daily dose increased slowly with time. Use of magnesium or aluminium-containing binders was negligible.

**Table 2:** Trends in average medication dose per day among users

**Year**	**Calcium**	**Sevelamer**	**Lanthanum**	**All phosphate binders**	**Cinacalcet**	**Alfacalcidol**	**Calcitriol**
	**(mg/d)**	**(mg/d)**	**(mg/d)**	(mg/d)*	**(mg/d)**	**(mcg/d)**	**(mcg/d)**
2001	1869	2991		2155		0.27	0.26
2002	1779	3477		2443		0.26	0.26
2003	1619	3875		2620		0.26	0.26
2004	1498	4030		2777		0.28	0.27
2005	1408	4312		2925		0.28	0.26
2006	1365	4364		2992	50	0.29	0.26
2007	1348	4331	1613	3013	50	0.31	0.25
2008	1329	4172	2433	3040	52	0.32	0.24
2009	1302	4078	2111	3166	55	0.35	0.24
2010	1265	4011	2074	3270	54	0.36	0.25

* All phosphate binders dose is given in mg of calcium phosphate-binding equivalent using relative phosphate-binding coefficients: 1.0 for calcium carbonate, 0.7 for sevelamer, and 2.0 for lanthanum [18].

As previously mentioned, before provincial reimbursement of cinacalcet in June 2006, some patients were receiving cinacalcet from Amgen. According to Amgen internal data, 4 patients received cinacalcet through this program in 2004, 40 in 2005, and 58 in 2006. Some private insurance companies may have reimbursed cinacalcet for patients not covered by the provincial RAMQ drug plan. Unfortunately, the exact number of patients receiving cinacalcet via private insurance is not available in our database. According to Amgen data, cinacalet claims were estimated at 241 in 2005 and 385 in 2006 (Amgen internal data). Assuming monthly claims (12 claims per patients per year), it can be estimated that 20 patients received cinacalcet through private insurance companies in 2005 and 32 patients in 2006.

## Discussion

In this large population-based study, a significant drop in PTX rates among patients receiving dialysis after January 1, 2006 is shown. This drop coincides with cinacalcet introduction, but an increasing trend in the use of phosphate binders (but less calcium-based binders) and vitamin D across the time period is also shown.

Our results are consistent with a pooled analysis of short duration trials that showed a reduce incidence of PTX with cinacalcet (from 41 /1000p-y in the placebo group to 3 /1000 p-y in the cinacalcet group) [3]. More recently, a large placebo-controlled trial designed to evaluate cardiovascular endpoints showed a 69% decreased risk of PTX in the cinacalcet group [4]. Few studies have evaluated trends in PTX rates, and only one has reported rates after the release of the KDOQI clinical practice guidelines in 2003, and none have included patients well beyond introduction of calcimetics [5-8]. Li et al. [5] described PTX rates in the US from 1992 to 2007. A PTX peak rate was found in 2002 (12.8 /1000 py) followed by a decreasing rate until 2005 (5.4 /1000 py) and then an increase again in 2006–2007 (8.6 /1000 py) [5]. The current data shows a different pattern where the peak annual PTX rate was in 2004 (16.0 /1000 py) instead of 2002, followed by a decrease in 2006 (3.9 /1000 py). After 2006, annual PTX rates remained between 3.0 and 4.6 /1000 py. While January 2006 was the only statistically significant cut-off point in our model, visually the change in rate appeared slightly before that and all cut-off dates between January 2005 and June 2006 showed a trend for a lower PTX rates. This is consistent with the fact that cinacalcet market penetration was progressive over that period of time, and the lack of statistical power may explain why most were not statistically significant. Despite not being widely available before June 2006, carefully selected patients could have received cinacalcet from Amgen or private insurance companies. In the context where cinacalcet was probably given to patients who would otherwise have undergone a PTX and because the annual counts of PTX ranged from 29 to 57 before 2005, only a few patients receiving cinacalcet could have highly influenced the observed PTX rates. For this reason, it is not unexpected to see a flat trend for PTX rates after 2006 while cinacalcet market share continued to increase constantly. Because severe secondary hyperparathyroidism may not be completely controlled in all patients using cinacalcet and because some patients may not tolerate cinacalcet or would prefer to undergo a PTX, it is expected that rates of PTX would not fall indefinitely. It is unknown why PTX rates did not increase in 2006–2007 as was observed in the US. However, it is speculated that wider availability of cinacalcet through reimbursement in Québec may be a factor. It is also possible that, despite the KDOQI guidelines opinion-based recommendation for PTX in patients “with severe hyperparathyroidism (persistent serum levels of intact PTH >88.0 pmol/L [800 pg/mL]), associated with hypercalcemia and/or hyperphosphatemia that are refractory to medical therapy” [19], Canadian physicians may be more inclined to wait for symptomatic hyperparathyroidism before sending a patient for a PTX. The higher PTX rates observed here among prevalent and younger patients are consistent with previous studies and with the physiopathology of secondary hyperparathyroidism [5,20].

The portrait of phosphate binders and vitamin D use has changed in the last decade. This data shows a clear shift to lower dosing of calcium-based binders and an increasing utilization of non-calcium-based binders such as sevelamer and lanthanum. While the average dose of vitamin D remains fairly low (alfacalcidol increased slightly and calcitriol remained stable), the proportion of prescribed vitamin D increased considerably, reaching 70% in 2010. Unfortunately, only intravenous vitamin D analogues were available in Li et al. [5] study, therefore use of phosphate binders, cinacalcet and oral vitamin D was not reported. Nevertheless, this switch from calcium-based to non-calcium based phosphate binders is expected after the publication of studies associating high calcium-phosphorus product with a higher risk of mortality and cardiovascular calcifications [21,22]. In 2003, KDOQI guidelines [19] recommended a maximum dose of 1500 mg/day of elemental calcium, which was significantly lower than the usual practice. The increased of phosphate binders and vitamin D within this RAMQ data is also probably explained by the impact of KDOQI Guidelines on clinical practice and targets for serum phosphorus and PTH, and initial vitamin D dose. Additionally, cinalcalcet reimbursement was conditional on specific criteria as detailed previously, and physicians were encouraged to use vitamin D and phosphate binders to gain access to cinacalcet for their patients.

This study reports on changes of PTX trends over time, but cannot establish a causal link between cinacalcet and lower PTX rates. A stronger design would have been to compare PTX rates in patients receiving cinacalcet with rates in patients who did not. However, in the absence of available PTH levels, it was not possible to identify a control group that would have had the same severity of hyperparathyroidism and did not receive cinacalcet. Therefore, results would have suffered from an indication bias. Change in rates may be due to other factors occurring simultaneously with the introduction of cinacalcet. For example, following the publication of the KDOQI Guidelines use of more aggressive treatments such as higher doses of phosphate binders and vitamin D may explain lower PTX rates with time [1,19]. Decreased use of calcium-based phosphate binders may also explain decreased PTX rates by decreasing serum calcium, which would allow increase vitamin D use. Another explanation would be a change of indication or physician attitude toward PTX. For example, physicians may now tolerate higher PTH level thresholds before considering a PTX. However, these clinical practice changes are usually not acute in time and cinacalcet introduction remains the most probable explanation for the reduction in PTX rates observed. Despite the fact that the drop in PTX rates is visually convincing during the intervention period, we tested six different intervention dates and statistically significant results may be due to chance by multiple testing (limited power precluded applying correction factors such as Bonferroni). The unadjusted PTX rates are presented, since small numbers precluded standardization. An aging population may lead to lower PTX rates while an increasing proportion of prevalent patients may increase PTX rates. However, the population characteristics did not change much during the study years and the same PTX trend pattern was seen among subgroups (Figure 3). Moreover, adjusting PTX rates in the Li et al. [5] study did not make a significant difference in the time period of the current study.

Some PTX procedures may have been missed if they were not coded. However, claims are required from surgeon compensation and it would be unlikely that a PTX would have been missed. RAMQ codes for PTX did not change during the study period. If any percutaneous ethanol ablation procedures of the parathyroid were carried out, these would not be captured, as there is no specific code for this new technique. But this technique was carried out on one or two anecdotal cases only and cannot explain the reduced rates. As with most retrospective studies using dispensed medications, there is uncertainty with patient compliance. In addition, intravenous vitamin D analogue claims are not captured within the RAMQ database, since vitamin D analogues are dispensed by the dialysis unit and a claim would not be submitted by the patient to the RAMQ. However, intravenous vitamin D is not commonly used in Québec, in part for economic reasons and health care organization. Finally, medication use among patients covered by private drug plans who are generally younger patients (an important risk factor for severe secondary hyperparathyroidism) could not be assessed. However, the main analysis on PTX trends included all patients regardless of age and availability of private insurance. Moreover, because the ARIMA model relied on time points and not on patient-level exposure, incomplete ascertainment of exposure does not influence our results. Finally, this study is limited to a single Canadian province, and results may not be generalized to other populations with different healthcare practices.

## Conclusions

A decreased annual rate of PTX was found after January 2006, corresponding to cinacalcet availability. However, decreased rates may be due to other factors occurring simultaneously with cinacalcet introduction and further studies are needed to confirm these findings.

## Competing interests

This work was supported by an unrestricted grant from Amgen Canada. Dr. Lafrance is supported by a KRESCENT New Investigator Award (public funding) and has received advisory-board and lecture fees from Amgen Canada for unrelated work. Dr. Le Lorier has received consultancy fees and grant support from Amgen Canada.

## Authors’ contributions

This manuscript is co-authored by J-PL (design, analysis, interpretation of data, and drafting the article), HC (conception and revising the article), ML (conception and revising the article), FM (conception and revising the article), VP (conception and revising the article), LR (conception and revising the article), and JLL (conception, interpretation of data, and revising the article). All authors have approved the final version.

## Pre-publication history

The pre-publication history for this paper can be accessed here:

http://www.biomedcentral.com/1471-2369/14/100/prepub
